# Space–Time Covariation of Mortality with Temperature: A Systematic Study of Deaths in France, 1968–2009

**DOI:** 10.1289/ehp.1307771

**Published:** 2015-03-23

**Authors:** Nicolas Todd, Alain-Jacques Valleron

**Affiliations:** U1169, INSERM (Institut national de la santé et de la recherche médicale), Le Kremlin–Bicêtre, France

## Abstract

**Background:**

The temperature–mortality relationship has repeatedly been found, mostly in large cities, to be U/J-shaped, with higher minimum mortality temperature (MMT) at low latitudes being interpreted as indicating human adaptation to climate.

**Objectives:**

Our aim was to partition space with a high-resolution grid to assess the temperature–mortality relationship in a territory with wide climate diversity, over a period with notable climate warming.

**Methods:**

The 16,487,668 death certificates of persons > 65 years of age who died of natural causes in continental France (1968–2009) were analyzed. A 30-km × 30-km grid was placed over the map of France. Generalized additive model regression was used to assess the temperature–mortality relationship for each grid square, and extract the MMT and the RM25 and RM25/18 (respectively, the ratios of mortality at 25°C/MMT and 25°C/18°C). Three periods were considered: 1968–1981 (P1), 1982–1995 (P2), and 1996–2009 (P3).

**Results:**

All temperature–mortality curves computed over the 42-year period were U/J-shaped. MMT and mean summer temperature were strongly correlated. Mean MMT increased from 17.5°C for P1 to 17.8°C for P2 and to 18.2°C for P3 and paralleled the summer temperature increase observed between P1 and P3. The temporal MMT rise was below that expected from the geographic analysis. The RM25/18 ratio of mortality at 25°C versus that at 18°C declined significantly (*p* = 5 × 10^–5^) as warming increased: 18% for P1, 16% for P2, and 15% for P3.

**Conclusions:**

Results of this spatiotemporal analysis indicated some human adaptation to climate change, even in rural areas.

**Citation:**

Todd N, Valleron AJ. 2015. Space–time covariation of mortality with temperature: a systematic study of deaths in France, 1968–2009. Environ Health Perspect 123:659–664; http://dx.doi.org/10.1289/ehp.1307771

## Introduction

The variation of daily mortality with the temperature at the time of death has repeatedly been found to be U/J-shaped, with increased mortality at both low and high temperatures (Rau 2007). Generalized additive models (GAM) (Hastie and Tibshirani 1990; Wood 2006) provide a powerful framework to model the temperature–mortality relationship and estimate its minimum [sometimes referred to as the minimum mortality temperature (MMT)], while taking into account potential confounding factors (Curriero et al. 2002).

Comparative studies at different latitudes found repeatedly that the temperature at which mortality was minimal was higher in places where the mean summer temperature (MST) was higher. For example, the authors of an observational study in seven different European settings (Keatinge et al. 2000) found that the observed 3°C-wide band with the lowest mortality ranged from 14.3–17.3°C in north Finland, to 19.3–22.3°C in London, and to 22.7–25.7°C in Athens. In 11 eastern U.S. cities, the MMT correlated with the latitude (Curriero et al. 2002). Similar results were obtained in Spain, where 13 cities were compared (Iñiguez et al. 2010). One interpretation of the variation of the MMT with temperature of the place for which it is computed, as of other parameters [e.g., excess winter mortality, found to be highest in countries with milder winters (McKee 1989)], is that human behaviors adapt to local climatic conditions (Keatinge et al. 2000; Rau 2007). Drawing conclusions from this geographic observation about the possible adaptability of human populations to future climate change requires observing that, similarly, MMT at a given location changes over time when climate changes.

Herein, we report our analysis of the temperature–mortality relationship in continental France during the 42 years between 1968 and 2009. Continental France is approximately 900 km × 900 km, and exhibits broad climatic diversity (Laaidi et al. 2006). The study covered a period of warming in France and worldwide (National Aeronautics and Space Administration Goddard Institute for Space Studies 2015). Our primary goal was to describe the temperature–mortality relationship at the highest spatial resolution possible to include small towns and rural settings that have so far been poorly investigated. The second goal was to determine whether MMT increased in parallel to the MST rise observed in France over the last four decades.

## Methods

*Climate data*. We used the ENSEMBLES gridded observational data set (E-OBS) (Haylock et al. 2008; van den Besselaar et al. 2011), which provides the daily mean temperatures, precipitations, and average sea-level pressures in Europe with a 0.25°-latitude × 0.25°-longitude resolution.

**Figure 1:**
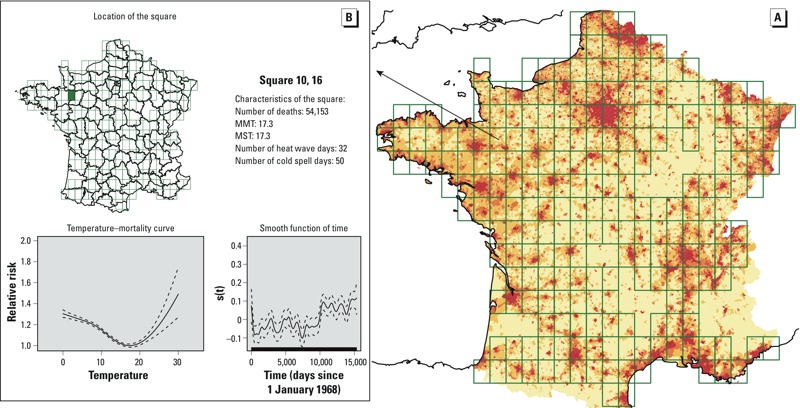
(*A*) Grid placed over continental France to obtain the squares used in the analysis. The pixel color indicates the population density in 2008 (red: highest; pale yellow: lowest). The temperature–mortality analysis (shown in (*B*) for a particular square in the western part of France) was done for each of the squares with > 1.5 deaths/day during each of the studied periods, corresponding to 22,500 deaths in the total analysis (228 squares), and to 7,500 deaths/period for the by-period analyses (224 squares). For each grid square, a GAM was used to model the temperature–mortality relationship and extract the corresponding curve and three parameters: the minimum mortality temperature (MMT) and the relative risk of mortality expressed as the ratios of mortality observed at 25°C to that observed at MMT (RM25) or that observed at 18°C (RM25/18). This map is interactive at http://www.isis-diab.org/isispub/ehp/interactive_map_wp.htm. (*B*) Clicking on any square of the map shows vital information on the square (top), the smooth function of time [s(t) (defined in Equation 1) bottom right], and the variation with temperature of the relative risk of total mortality for individuals > 65 years old [the reference risk was the mortality observed at the MMT of each square: here (bottom left) is thus plotted *s*_1_(T)/*s*_1_(MMT)]. One example is shown here: The detailed information pertaining to the 1968–2009 period for all squares of the grid is obtained on the interactive map where the temperature–mortality curves can be viewed for the all the study periods [P_all_ (1968-2009), P1 (1968–1981), P2 (1982–1995), and P3 (1996–2009)].

*Discretization of space*. The temperature–mortality relationship was studied within each 0.50°-latitude × 0.50°-longitude “square” (approximately 30 km × 30 km) of a grid placed over the map of continental France. This grid was built by aggregating four 0.25°-latitude × 0.25°-longitude squares of the E-OBS data set, starting at 40.625° latitude and –5.625° longitude and ending at 10.625° latitude and 51.875° longitude, yielding 295 squares (Figure 1), for which the daily mean temperatures, precipitations, and average sea-level pressures were obtained by averaging the four 0.25°-latitude × 0.25°-longitude E-OBS database source values.

The smallest administrative territorial partition of France is the “commune” [the French version of the second level of Local Administrative Units (LAU 2) in the nomenclature of the European Union (Eurostat 2015)]. Sociodemographic data used herein are available at this level. There are roughly 36,000 communes in continental France, with wide variability in terms of surface and population. Each of the communes was attached to the 0.50°-latitude × 0.50°-longitude square of the grid to which its centroid belonged. Because administrative changes (merger of two communes, separation of one commune in two new communes, creation of a new commune) occur frequently, we curated the original database to take into account that communes could appear or disappear over the 42-year study period. For example, in the final database, two communes that merged at a date within the study period were considered merged for the entire period and attributed a single centroid.

*Periods considered in the analysis*. Two analyses of the temperature–mortality relationship were done. In the first analysis, the whole 42-year period (P_all_) was considered. The temperature–mortality relationship was evaluated for each grid square (*n* = 228) with > 22,500 deaths during 1968–2009 (approximately 1.5 deaths/day). In the second analysis (“by-period” analysis) three equal 14-year periods were considered: 1968–1981 (P1), 1982–1995 (P2), and 1996–2009 (P3). The temperature–mortality relationship was examined for each grid square with > 7,500 deaths during each of the three periods, corresponding to 224 squares. Finally, a sensitivity analysis of the by-period analysis concerned squares in which the minimum number of deaths had to be > 15,000, > 22,500, or > 37,500 (respectively, 3, 4.5, or 7.5 deaths/day), concerning respectively, 99, 47, and 15 squares.

*Mortality data*. These data were the individual death certificates of persons > 65 years old who died in continental France between 1968 and 2009. They were collected and curated by the French Center for Epidemiology of Mortality Data (CépiDc 2015). The National Commission on Information Technology and Liberties [Commission nationale de l’informatique et des libertés (CNIL) 2015] granted us authorization to access and analyze those data.

From the death certificates, we obtained the date of birth, the date of death (precision: day), the permanent residence before death (precision: commune), and the underlying cause of death. The death certificates for 1968–1978, 1979–1999, and 2000–2009, respectively, were coded according to the 8th, 9th, and 10th revisions of the *International Classification of Diseases* (ICD) (World Health Organization 2015). We excluded from the analysis death certificates with an underlying cause of death coded as “external” (ICD-8 and ICD-9: > E800; ICD-10: V01–Y89), except for those possibly linked to climate (“natural or unknown sources of heat or cold”: ICD-8 and ICD-9: E900.0, E900.9, E901.0, E901.8, E901.9; ICD-10: X30 and X31). Therefore, all natural causes, with the above-mentioned exception, were thus included, for a total of 16,487,668 deaths.

*Sociodemographic data*. Sociodemographic data was used to assess potential explanatory variables of MMT. The population density, the unemployment rate for age group 25–54 years and the proportion of secondary school graduates (12th grade) in the population > 25 years old were obtained from the French National Institute of Statistics and Economic Studies (Insee 2015), for each commune at census years (1968, 1975, 1982, 1990, 1995, 2008). In the by-period analysis, we calculated the means for 1968 and 1975, 1982 and 1990, and 1995 and 2008 to characterize the sociodemographic status during periods P1, P2, and P3, respectively. Because the source data on communes were rates (e.g., unemployment rate), we searched for the original additive variables to obtain the rates for each grid square (e.g., absolute numbers of unemployed persons and total labor force) by summing the corresponding absolute values for all communes whose centroid was within the square. The infant-mortality rates (number of deaths < 1 year old per 1,000 live births) at the square level were computed from birth data (Insee 2015) and mortality data for the cohorts born in 1968–1981, 1982–1998, and 1999–2007.

*Statistical analyses*. For each grid square and for each period (P_all_, P1, P2, P3), we fitted a GAM:

log[*E*(*Y_t_*)] = *s*_1_(*T_t_*) + *s*_2_(*R_t_*) + *s*_3_(*P_t_*) + *s*_4_(*t*) + α*Hw* + β*Cs* + γ*Dow,* [1]

where *Y_t_* is the death count on day *t*, assumed to be Poisson-distributed around *E*(*Y_t_*); *s*_1_, *s*_2_, *s*_3_, and *s*_4_ are penalized cubic regression splines modeling the contribution of temperature *T_t_*, sea-level pressure *P_t_*, and precipitations *R_t_*; and *s*_1_ is chosen with 5 degrees of freedom (df), *s*_2_ and *s*_3_ with 3 df, and *s*_4_ with 14 df (that is, 1 df/year). The knots are set at the distribution quantiles corresponding to the df chosen for each variable. The smoothing parameter controlling the penalization of the spline functions is chosen to minimize the unbiased risk estimator score (Wood 2006). *Hw* and *Cs* are dummy variables for a heat wave (Hajat et al. 2006) or a cold spell. A heat-wave or cold-spell day is defined, respectively, as being at least the 4th day above the 99th or below the 1st percentile of the local temperature distribution. *Dow* is the day of the week.

*Abstractions of the temperature–mortality curves*. To summarize the temperature–mortality curves, we extracted four parameters for each of the regressions. The curves were classified according to their U/J shape (yes/no) on the 0°–30° range of mean daily temperatures (see Figure 1 for an example of the curves generated for one square). The curves generated for each square are available on an interactive map (Figure 1 shows a static version; see http://www.isis-diab.org/isispub/ehp/interactive_map_wp.htm, “Whole-period analysis,” to access the active version of the map). The MMT of U/J-shaped curves, as defined by Curriero et al. (2002), was extracted from the regression model. It was defined as the minimum of *s*_1_ on the 0–30°C range (precision: 0.1°C). Finally, the estimated ratio of mortality (RM) at 25°C compared with that at MMT {RM25 = exp[*s*_1_(25) – *s*_1_(MMT)]}, and the estimated ratio of mortality at 25°C compared with that at 18°C {RM25/18 = exp[*s*_1_(25) – *s*_1_(18)]}, were computed. In the by-period analysis, Wilcoxon signed-rank tests were used to compare the changes in mean MMT, RM25, and RM25/18.

*Geographic structure*. Moran’s index (Gaetan and Guyon 2010; Moran 1950) was computed to assess the geographic structure of MMT and the spatial autocorrelation potentially left in regression residuals. To calculate this index, the weights were taken to be identical to those used for the spatial interpolation (see above). Standard statistical R-packages (R Core Team 2013) were used to investigate the correlations between MMT and meteorological or socioeconomic characteristics of each grid square, and during each study period.

Spatial interpolation was used to visualize the geographic MMT variations. The value *MMT_est_*(*z*) interpolated at position *z* on the map was given by

*MMT_est_*(*z*) = ^[^Σ*__i__w*(*c_i_,z*)*MMT_est_* (*c_i_*)^]^/Σ*__i__w*(*c_i_,z*), [2]

where *c_i_* is the position of the center of the *i*th square yielding an MMT, and *w*(*c_i_*,*z*) = (*c_i_* – *z*)^–2^ is the weight, taken to be equal to the inverse squared distance between *c_i_* and *z*.

*Sensitivity analysis*. We tested the sensitivity of our results to the df/year value chosen with two additional analyses: in the first one (see Supplemental Material, “Model 1: 2df/y”), we used 2 df/year. In the second sensitivity analysis (see Supplemental Material, “Model 2: 4df/y and lagged temperature”), we used 4 df/year, and in addition we modeled the lagged effect of temperature on mortality by adding to the regression model the mean temperature for the preceding 6 days. This second additional model was thus on each square:

log[*E*(*Y_t_*)] = *s*_1a_(*T_t_*) + *s*_1b_(*T_t_*_– 1;_*_t_*_– 6_) + *s*_2_(*R_t_*) + *s*_3_(*P_t_*) + *s*_4_(*t*) + α*Hw* + β*Cs* + γ*Dow,* [3]

with *s*_4_ modeled with 4 df/year (168 df at total). MMT was defined as the minimum of *s*_1a_ + *s*_1b_ (see Supplemental Material, Figure S2, for an example). In this analysis, the data of the whole 42 years period were used.

All analyses were performed in R, with package MGCV for GAM regressions (Wood 2015) and SP for spatial interpolation (Bivand et al. 2008)

## Results

**Figure 2:**
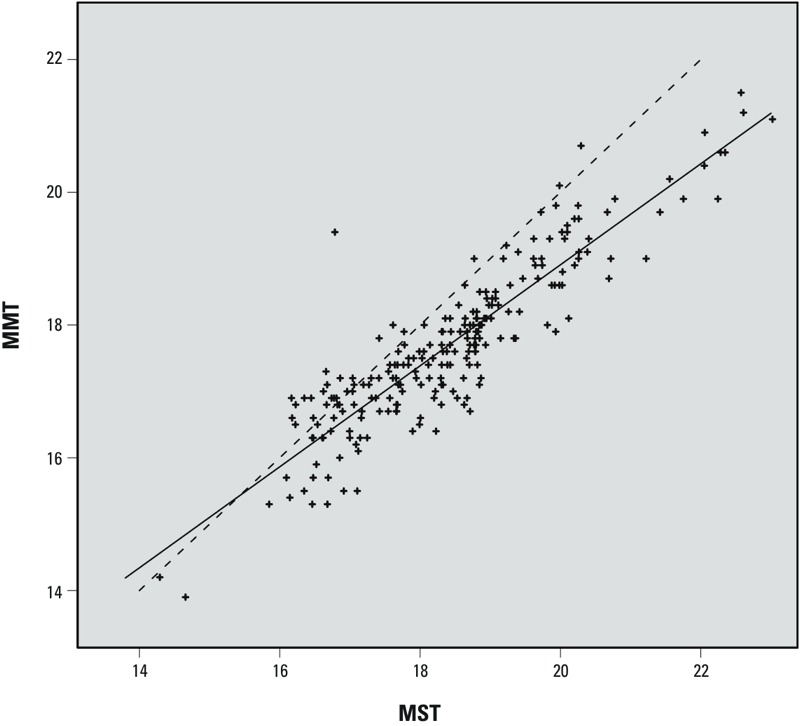
Geographic variations of MMT versus MST in France. Each point represents the MMT and MST values in one of the 228 grid squares shown in Figure 1, during the study period 1968–2009. The squares analyzed are those with > 22,500 deaths during the study period (~ 1.5 deaths/day). Solid line: regression line. Dotted line: MMT = MST.

*The temperature–mortality relationship in France: 1968–2009*. The temperature–mortality relationship observed for each of the 228 squares analyzed when all 42 years of data (15,341 days) were considered was, without exception, U/J-shaped (as shown in the interactive map “Whole-period analysis” at http://www.isis-diab.org/isispub/ehp/interactive_map_wp.htm). The MMT values were strongly correlated to latitude (*r* = –0.63, *p* < 2.2 × 10^–16^), MST (*r* = 0.9, *p* < 2.2 × 10^–16^), and mean winter temperature (MWT) (*r* = 0.62, *p* < 2.2 × 10^–16^). The MST–MMT relationship was linear (Figure 2). A 1°C increase in MST corresponded to a 0.76°C [95% confidence interval (CI): 0.71, 0.81] increase in MMT.

**Figure 3:**
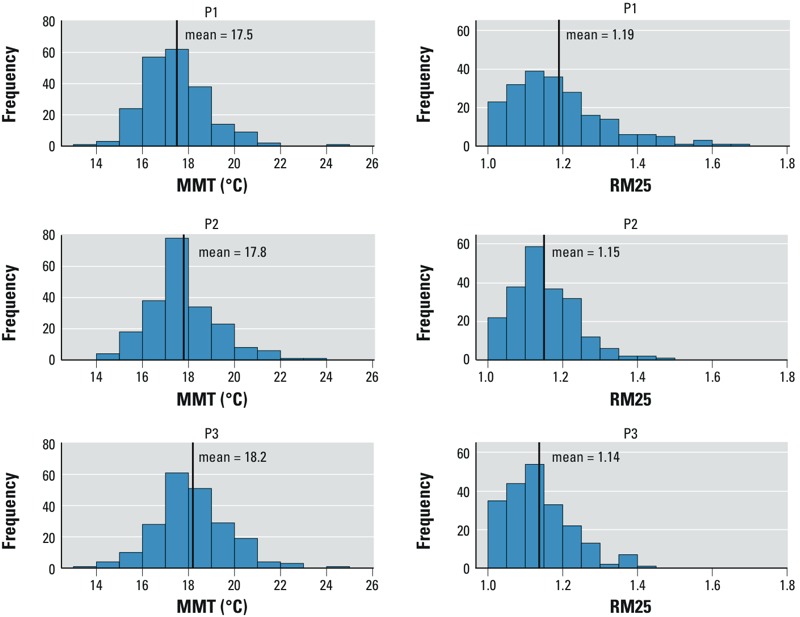
Distributions of minimum mortality temperatures (MMT) and relative risk of mortality expressed as the ratios of mortality observed at 25°C to that observed at MMT (RM25), computed for three successive periods in France (1968–2009).

*The temperature–mortality relationship in France during three successive 14-year periods*. A U/J-shaped curve was found for all three periods in 211 (94%) of the 224 squares (see By-period analysis: “Map 1. Cut off at 7,500*”* at http://www.isis-diab.org/isispub/ehp/interactive_map_co75.htm). The global MMT distribution for those 211 squares with U/J-shaped curves shifted to higher temperatures over time. For P1, P2, and P3, respectively, the values at the 25th percentile were 16.4°C, 16.8°C, and 17.2°C, and the values at the 75th percentile were 18.2°C, 18.6°C, and 19.3°C. The mean MMT rose from 17.5°C to 17.8°C and 18.2°C, and the whole MMT distribution shifted toward higher temperatures with time (Figure 3). This increase paralleled that of MST (+1.5°C between P1 and P3) and MWT (+0.8°C between P1 and P3) (Table 1). RM25 and RM25/18 shifted to lower values with time (Figure 3). The mean values of RM25 and RM25/18 (Table 1) were significantly lower at P3 than at P1 (*p*-values: 10^–7^ for RM25; 5 × 10^–4^ for RM25/18). The unemployment rate was the only sociodemographic variable significantly correlated with MMT for each of the three periods (Table 2).

**Table 1:** Climate and mortality characteristics during the three periods analyzed (mean ± SD).

Period	MST (°C)	MWT (°C)	Heat-wave days	Cold-spell days	MMT (°C)	Days > MMT days	RM25	RM25/18
P1: 1968–1981	17.6 ± 1.4	4.2 ± 1.8	6.8 ± 6.0	8.2 ± 2.4	17.5 ± 1.4	825 ± 228	1.19 ± 0.13	1.18 ± 0.12
P2: 1982–1995	18.6 ± 1.5	4.6 ± 1.8	5.8 ± 4.0	22.6 ± 3.5	17.8 ± 1.5	962 ± 243	1.15 ± 0.09	1.16 ± 0.08
P3: 1996–2009	19.2 ± 1.5	5.0 ± 1.7	16.7 ± 5.5	8.6 ± 2.9	18.2 ± 1.6	994 ± 262	1.14 ± 0.09	1.15 ± 0.08
Abbreviations: MMT, minimum mortality temperature; days > MMT, at a given period, sum on all squares of the number of days observed with a temperature above the MMT of the square at the given period; RM25, ratio of mortality at 25°C/mortality at MMT; RM25/18, ratio of mortality at 25°C/mortality at 18°C. Results were computed in the 211 squares with > 7,500 deaths and a U/J-shaped curve for all three periods. Mean summer temperature (MST) and mean winter temperature (MWT), respectively, are the mean temperatures observed during the months of June–August and December–February of the period considered.								

**Table 2:** Correlations between minimum mortality temperatures (MMT) and local sociodemographic characteristics.

Period	Population density	Unemployment rate	Proportion of secondary school graduates	Infant mortality rate
P1: 1968–1981	–0.01 (*p *= 0.89)	0.30 (*p *= 10^–5^)	0.13 (*p *= 0.06)	–0.01 (*p *= 0.89)
P2: 1982–1995	0.00 (*p *= 1)	0.30 (*p *= 10^–5^)	0.10 (*p *= 0.15)	0.11 (*p *= 0.11)
P3: 1996–2009	0.00 (*p *= 1)	0.26 (*p *= 10^–4^)	0.03 (*p *= 0.66)	0.12 (*p *= 0.08)
The correlations were computed between the estimated MMT and the sociodemographic characteristics of the 211 grid squares with > 7,500 deaths during each of the three study periods and a U/J-shaped curve for each period. Correlations were assessed with Pearson’s correlation coefficient. *p*-Value indicates the significance level of the test of independence.				

**Figure 4:**
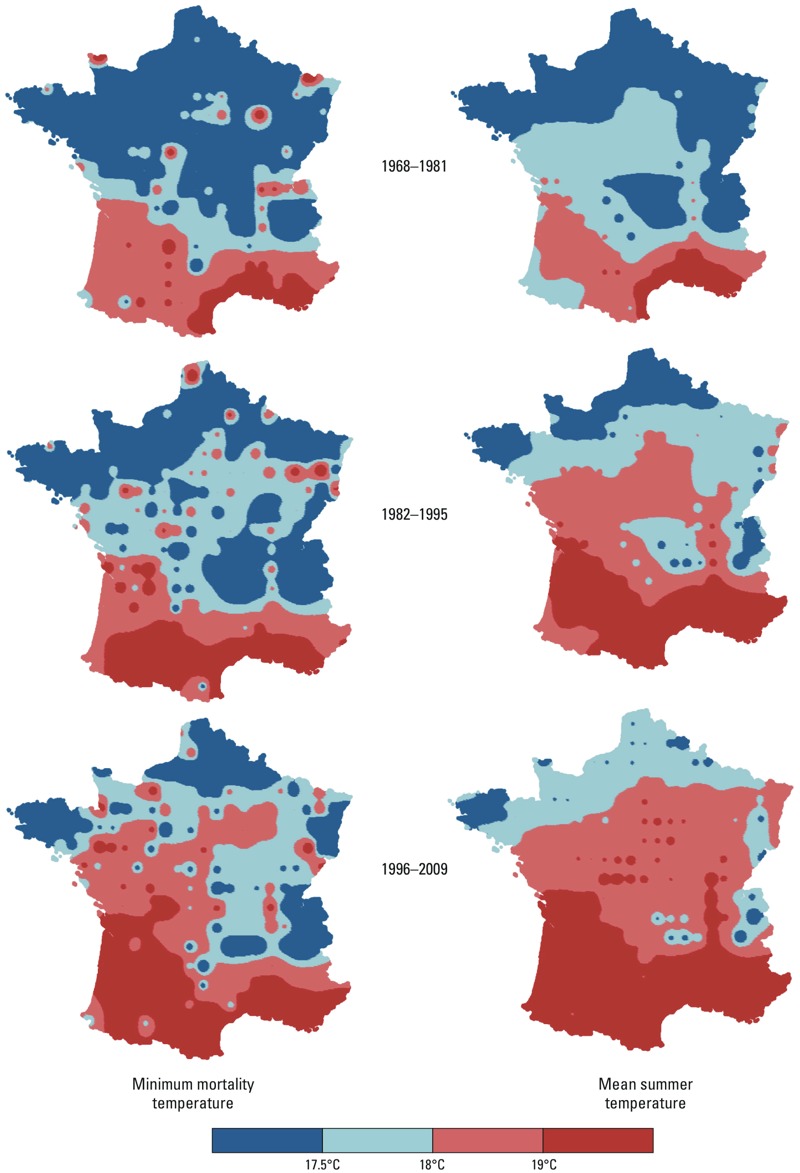
Variations of minimum mortality temperatures (MMT) and mean summer temperatures (MST) in France from 1968 through 2009. The maps were interpolated from the values observed at the centroids of the 211 grid “squares” with U/J-shaped curves at the three successive 14-year periods (see “Methods” for details on the smoothing technique used).

Moran’s index computed for the MMT of the 211 squares was 0.15 during P1, 0.14 during P2, and 0.13 during P3 (significant at the 5% level with the usual regularity assumptions on the random field). The geographic MMT pattern paralleled the geographic distribution of MST, with higher MMTs in the southern part of the country (Figure 4).

When the by-period analysis was restricted to the grid squares with > 22,500 or > 37,500 deaths/period (approximately 4.5 and 7.5 deaths/day, respectively), all squares had U/J-shaped curves for all three periods (see By-period analysis: Maps 2–4 at http://www.isis-diab.org/isispub/ehp/interactive_map_co150.htm). When the threshold was set at 15,000 deaths/period (approximately 3.0 deaths/day), 98 of the 99 squares were U/J-shaped at all three periods. Regardless of the threshold applied, the MMT distributions across the grid shifted similarly to higher temperatures with time. Mean MMT were 17.6°C for P1, 17.9°C for P2, and 18.1°C for P3 for the 98 squares with > 15,000 deaths; 17.4°C for P1, 17.9°C for P2, and 18.1°C for P3 for the 47 squares with > 22,500 deaths; and 17.5°C for P1, 17.9°C for P2, and 18.3°C for P3 for the 15 squares with > 37,500 deaths. Similar MMT rises with time were observed when we repeated our analysis within groups of squares sharing the same pattern of annual temperatures (16 groups were built by considering all combination of quartiles of mean winter temperature and of mean summer temperatures; results not shown).

In the sensitivity analysis, the “new” MMTs, obtained by the two alternative models, were hardly different from those obtained with Equation 1 (see Supplemental Material, Figures S1 and S3, for the “new” MMTs as functions of MMTs obtained with Equation 1). In the second alternative model, 3 of the 228 squares with more 22,500 deaths were not U/J-shaped.

## Discussion

The primary objective of this analysis was to study comprehensively the temperature–mortality relationship across space, and not to restrict the investigation to places defined administratively. Most of the previous studies addressed this relationship for large cities. Rural or semiurban locations were not examined, even though some of the factors influencing human response(s) to climate differ between large cities and low-density places (e.g., the availability of health resources, social support, air conditioning). In addition, large urban areas are not homogeneous. For example, the administratively defined “city” of Paris (2.3 million inhabitants) and its suburbs (the urban area of Paris has 12.2 million inhabitants in all) are very heterogeneous. Moreover, there are more similarities between the city of Paris and its southwestern suburbs than between its southwestern and northeastern suburbs (Fleury et al. 2012). These arbitrary administrative distinctions became our first reason to explore the geography of the temperature–mortality relationship with a grid having the highest resolution possible. A 30-km × 30-km grid was retained as a trade-off between high resolution and a sufficient number of deaths per day per square to perform the regression analysis. The second reason to choose a grid framework, rather than the more frequent analysis by administrative entities, is that it is also the most common format of public social and ecological databases. In this study, we used only one variable per broad category of vital information available on local societies (infant mortality as a marker of health services, unemployment as a marker of poverty, completion of high school as a marker of education, population density as a marker of urbanization). However, hundreds of local data are available to provide detailed information on sociodemographic, geographic characteristics of populations. The spatial resolution of the corresponding databases can be very high. For example, population-density figures are available in France and worldwide at 1-km × 1-km resolution (Dobson et al. 2000). Satellite photos yield high-resolution maps of different air-pollution measures, which still need to be validated on the ground, but are highly promising for large-scale climate–heath studies (Hidy et al. 2009). Increasingly, these “Big Data” are made available free-of-charge to researchers. Therefore, it will be soon possible to map the residence of individuals before death, perhaps even during different periods during each one’s lifetime, to a variety of possible cofactors of the temperature–mortality relationship. One limitation is the need to respect privacy; and, for example in this study, the residence before death was made available only at the commune level, which nonetheless provided sufficient precision given the spatial variation of the parameters we considered [there are 36,000 communes in the 550,000 km^2^ of France, which extrapolates to a mean surface of 15.3 km^2^/commune, to be compared with the surface of one elementary E-OBS square of our grid (900 km^2^)].

In our by-period analysis, we balanced the advantage of considering short periods and small geographic units, which make it possible to describe subtle spatiotemporal changes, and the need to have sufficient data in each of the arbitrary space × time divisions to model the temperature–mortality relationship. Because of France’s very wide geographic diversity of climate, we decided to use relatively small “squares,” built with just four elementary squares (i.e., squares for which the E-OBS data set gives climate variables). We decided to model the temperature–mortality relationship with the lowest possible threshold of cases per square, i.e., 1.5 deaths/year. However, even this low value, when extrapolated to the whole period to squares with > 22,500 deaths, remarkably yielded U/J-curves for each of the grid squares. In the by-period analysis, the numbers of deaths were proportionally smaller, and 13 of the 3 × 224 squares (i.e., < 2%) no longer had the U/J-curves seen in the complete analysis.

Using this grid approach, we showed that the temperature–mortality relationship was U/J-shaped in all locations, even low-density rural settings. As for earlier investigations conducted in other places, we found that MMT was highest in the southern parts of the country. When all 42 years were pooled in the same analysis, the correlation coefficient was 0.90. In other words, approximately 80% of MMT variance was accounted for by the MST variations over France, thereby explaining the striking parallelism between the spatial structures found for MMT and MST.

The MMT estimates are conditional to the model used to express the temperature–mortality relationship. An important parameter in the statistical method used was the choice of the number of df/year attributed to the time function. We chose to model this function with only 1 df. This was aimed at taking into account slow changes of population numbers and life expectancy in each square considered. We tested the sensitivity of our results to the df/year value chosen with two additional analyses (see Supplemental Material, “Model 1: 2df/y” and “Model 2: 4df/y and lagged temperature”), to take into consideration that the annual winter mortality peak is attributable not entirely to climate conditions but to other factors, such as influenza and its complications (Donaldson and Keatinge 2002; Keatinge et al. 2000; Reichert et al. 2004), and that cold temperatures may have a prolonged effect on mortality. Resulting MMTs obtained in these sensitivity analyses were hardly changed.

Our study of the temperature–mortality relationship over time covers, to our knowledge, the longest period ever studied: 42 years. During that period, the effect of climate warming was visible in France, with notable MST and MWT rises (Table 1). Between P1 and P3, mean MST has increased by 1.6°C while mean MMT has increased by 0.8°C. The size of this temporal increase can be evaluated by examining the results of the spatial analyses: From the spatial analysis at P1, one can infer that when one place had an MST > 1°C higher than another place, its MMT was, on average, 0.69°C (95% CI: 0.58, 0.79) higher. Applying this geographical result to the 1.6°C change in MST, the predicted MMT increase would be 1.6 × 0.69°C = 1.1°C (95% CI: 0.9°C, 1.3°C), while the observed MMT increase was 0.7°C. If we adhere to the often-expressed view that MMT is an indicator of human adaptation, one can conclude that there is an adaptation to climate change; but perhaps because it takes time to become established, this adaptation is smaller than that observed between different populations in different climates that adapted long ago to their geographic conditions. Also, the MMT rise with MST, as a consequence of climate change, is not the only parameter to consider to predict the public health impact of climate warming. A decrease of the ratio measuring the mortality at 25°C, compared with that at MMT (RM25), was also found. That diminution was not merely a consequence of MMT’s rise because RM25/18 declined similarly to RM25. Another indication of a possible population adaptation to climate change was also found by considering the time–space variation of the annual date of minimum mortality, taken as the date when the 11-day–centered moving average of the chronological series of mortality (results not shown) was the lowest: This date shifted toward the fall with period (24 August for P1, 2 September for P2, and 9 September for P3) and with latitude (2 September for the 140 grid squares north of the median French latitude, and 3 September for the 71 grid squares south of it).

The MMT increases and RM25, RM25/18 decreases that we have estimated are good news. Nevertheless, the numbers of days above the MMT increased importantly over the studied period (Table 1), which is worrying. On the other hand, the MWT increase can, more optimistically, lower the winter mortality peak. Hence, the impact of global warming on mortality is obviously multiparametric, and the parameters that we have studied are only pieces of the total puzzle.

New comprehensive spatiotemporal studies, using a design similar to this one, with the lowest resolution possible that is compatible with the requirements of the statistical estimation done and taking advantage of the mass of environmental public databases that are now increasingly available, should provide better understanding of the extent to which humans adapt to climate change, and the precise social and environmental conditions of this adaptation.

## Supplemental Material

(273 KB) PDF
