# Supplementary material for: Space–Time Covariation of Mortality with Temperature: A Systematic Study of Deaths in France, 1968–2009
Source: Environ Health Perspect. 2015 Mar 23;123(7):659–64. doi: 10.1289/ehp.1307771 (PMC4492259; doi:10.1289/ehp.1307771)
Supplement: (273 KB) PDF [file ehp.1307771.s001.acco.pdf]

Environ Health Perspect
doi: 10.1289/ehp.1307771

Note to Readers: ***EHP*** **strives to ensure that all journal content is accessible to all readers. However, some figures and Supplemental Material published in** ***EHP*** **articles may not conform to 508 standards due to the complexity of the information being presented. If you need assistance accessing journal content, please contact ehp508@niehs.nih.gov. Our staff will work with you to assess and meet your accessibility needs within 3 working days.**

Supplemental Material

Space–Time Covariation of Mortality with Temperature: A Systematic Study of Deaths in France, 1968–2009

Nicolas Todd[1] and Alain-Jacques Valleron[1,2]

Table of Contents

Sensitivity analyses

Model 1: 2df/y

Figure S1. Comparison of MMT obtained with 1 and 2 df/y for s(t) for the 211 squares with > 7,500 deaths and a U/J-shaped curve for all three periods. The full line figures the equality between the MMT estimates.

Model 2: 4df/y and lagged temperature

Figure S2. Derivation of the temperature-mortality relation with Model 2 in square 27-17 of the grid. Top panels: left $s_{1a}(T_t)$, right $s_{1b}(T_{t-1;t-6})$. Bottom panel: the final temperature-mortality curve $(s_{1a}(T)+ s_{1b}(T))/ (s_{1a}(MMT)+ s_{1b}(MMT))$.

Figure S3. Comparison of MMT estimates obtained with Model 2 (4df/year + lagged effect of temperature) with those presented in the paper. Two separate analyses were done: The left panel presents the results obtained on the squares with > 22,500 deaths and U/J curves (n=225). The right panel presents the results obtained on the squares with > 50,000 deaths and U/J squares (n=107) 107 U/J curves.

Sensitivity analyses

Model 1: 2df/y

Testing for the sensitivity to the degree of freedom allowed to the smooth function of time was conducted by fitting a model with 2 df/y for s(t) in the by period analysis (see also Model 2 where 4df/year were used)

As shown in the figure below, the new MMT values found at each of the 3 periods were close from the values obtained with the model with 1 df/year presented in the paper.

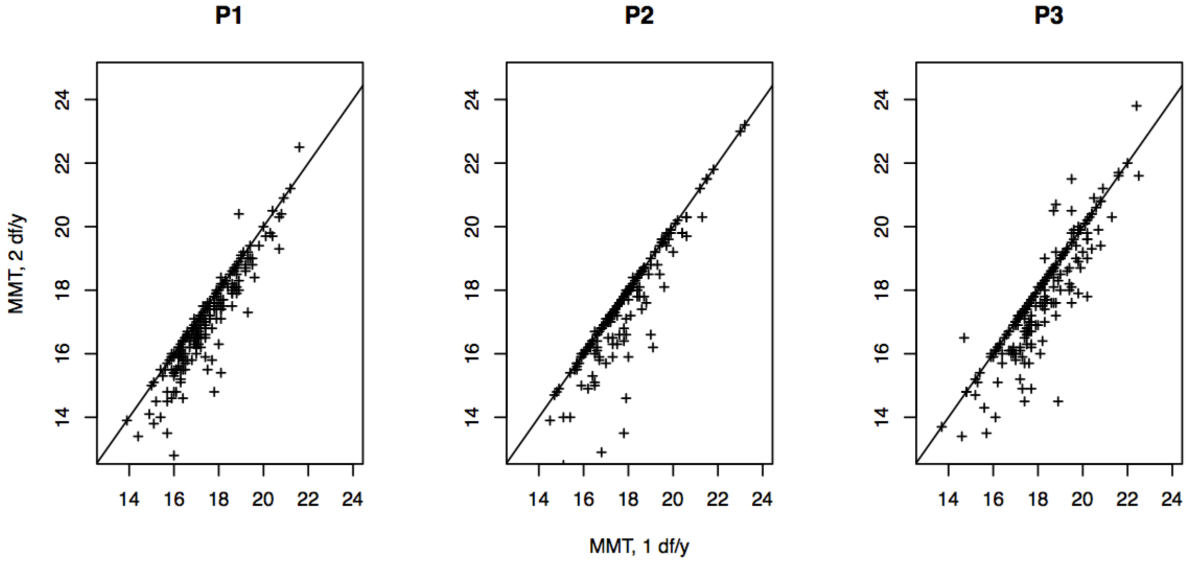

Figure S1. Comparison of MMT obtained with 1 and 2 df/y for s(t) for the 211 squares with > 7,500 deaths and a U/J-shaped curve for all three periods. The full line figures the equality between the MMT estimates.

Model 2: 4df/y and lagged temperature

In model 2, the possible lagged effects of temperature on mortality was adjusted for each square. The effects of T_t (temperature of the day) and $T_{t-1;t-6}$ (mean temperature over the past 6 days) were modelled separately.

One example of the estimation of the temperature-mortality relationship is shown on Figure S3.

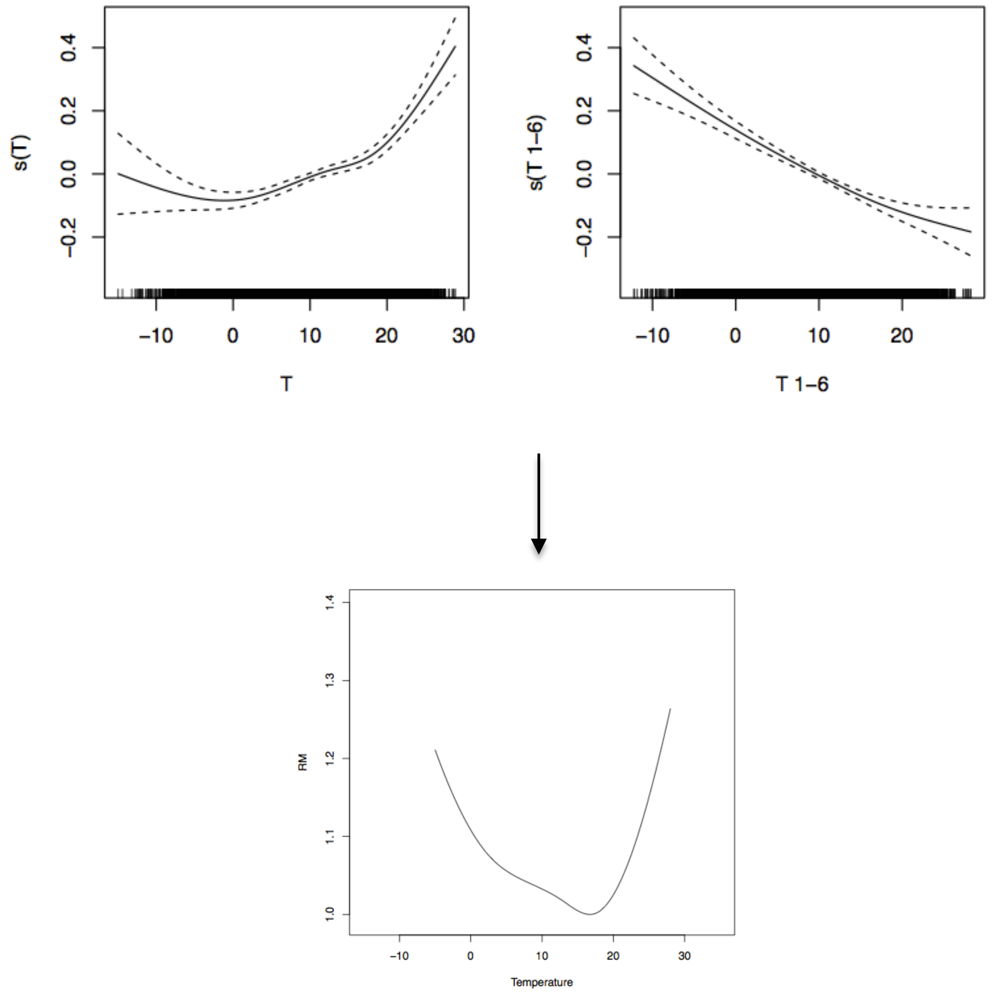

Figure S2. Derivation of the temperature-mortality relation with Model 2 in square 27-17 of the grid. Top panels: left $s_{1a}(T_t)$, right $s_{1b}(T_{t-1;t-6})$. Bottom panel: the final temperature-mortality curve $(s_{1a}(T)+ s_{1b}(T))/ (s_{1a}(MMT)+ s_{1b}(MMT))$.

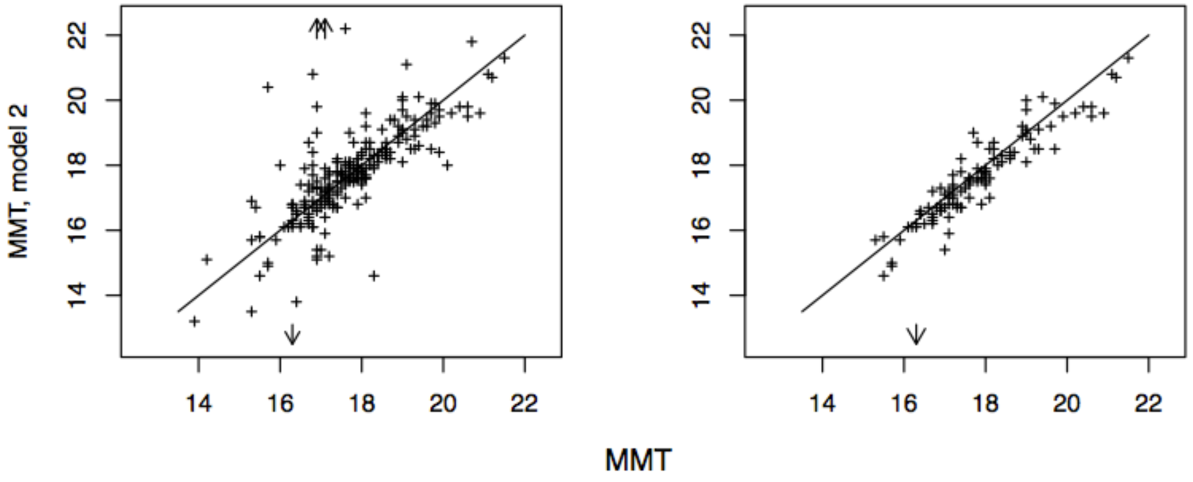

Figure S3. Comparison of MMT estimates obtained with Model 2 (4df/year + lagged effect of temperature) with those presented in the paper. Two separate analyses were done: The left panel presents the results obtained on the squares with > 22,500 deaths and U/J curves (n=225). The right panel presents the results obtained on the squares with > 50,000 deaths and U/J squares (n=107) 107 U/J curves.
